# Cerebellum as Initial Site of Distant Metastasis from Papillary Carcinoma of Thyroid: Review of Three Cases

**DOI:** 10.1155/2015/171509

**Published:** 2015-05-06

**Authors:** Mutahir A. Tunio, Mushabbab Al Asiri, Khalid Hussain AL-Qahtani, Wafa AlShakweer

**Affiliations:** ^1^Radiation Oncology, Comprehensive Cancer Center, King Fahad Medical City, Riyadh 59046, Saudi Arabia; ^2^Department of Otolaryngology-Head & Neck Surgery, College of Medicine, King Saud University, Riyadh, Saudi Arabia; ^3^Histopathology, Comprehensive Cancer Center, King Fahad Medical City, Riyadh 59046, Saudi Arabia

## Abstract

*Background*. The cerebellum as initial site of distant metastasis from differentiated thyroid carcinoma (DTC) including papillary (PTC) and follicular thyroid carcinoma (FTC) is rare manifestation. *Case Presentations*. Herein, we present three cases of cerebellar metastasis (CBM) of PTC. Mean age of patients was 67 years (range: 64–72), and mean duration between initial diagnosis and CBM was 49.6 months (range: 37–61). Frequent location was left cerebellar hemisphere and was associated with hydrocephalus. All patients underwent suboccipital craniectomy, and in two patients postoperative intensity modulated radiation therapy (IMRT) was given to deliver 5000 cGy in 25 fractions to residual lesions. Patient without postoperative IMRT had cerebellar recurrence along with lung and bone metastasis after 38 months. However, two patients were found alive and free of disease at the time of last follow-up. *Conclusion*. CBM from PTC is a rare clinical entity and is often associated with hydrocephalus. Histopathological diagnosis is important to initiate effective treatment, which relies on multidisciplinary approach to prolong the disease-free and overall survival rates.

## 1. Introduction

Differentiated thyroid carcinoma (DTC), especially papillary thyroid carcinoma (PTC), commonly metastasizes to regional lymph nodes, lungs, and bones. However, the brain is an uncommon site of metastasis, being found only in 0.15% to 1.3% of cases [1]. Moreover; cerebellar metastases (CBM) from PTC are exceptional; only thirteen reported cases of cerebellar metastasis from PTC have been published so far [2]. Symptoms of CBM may vary from asymptomatic to cerebellar dysfunction and stroke. CBM is considered as indicator of poor prognosis [3].

Herein, we describe and discuss the signs and symptoms, diagnostic work-up, differential diagnosis, and management in three cases of CBM from PTC.

## 2. Case 1

**Figure 1:**
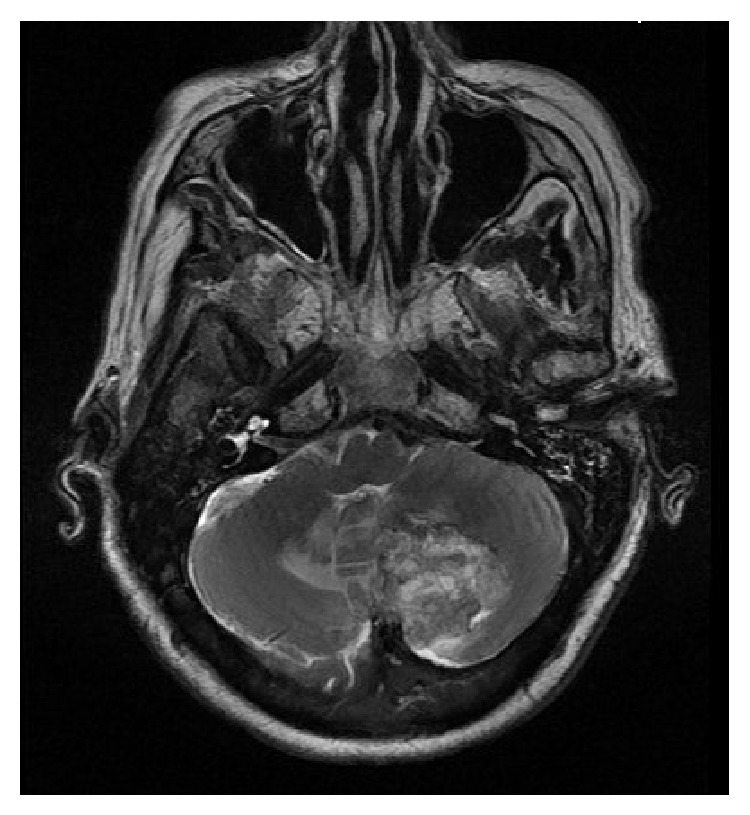
Magnetic resonance imaging of brain (axial view) showing a lobulated heterogeneous mass involving the medial left cerebellar hemisphere, measuring 4.0 × 3.5 × 2.5 cm associated with edema crossing the midline to the right cerebellar hemisphere, and compressing the fourth ventricle (the first case).

**Figure 2:**
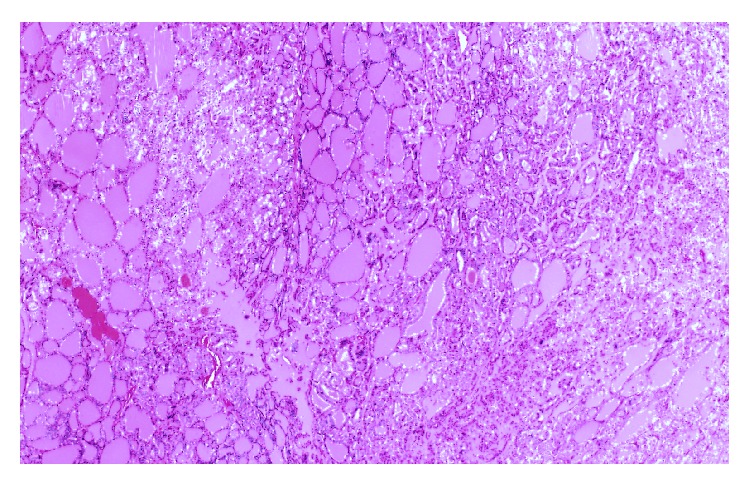
Hematoxylin and Eosin staining showing follicular pattern of papillary thyroid carcinoma (the first case).

A 72-year-old Saudi female known to be diabetic and hypertensive underwent total thyroidectomy, followed by radioactive iodine (RAI) ablation of 150 millicuries (mCi) under a diagnosis of PTC, follicular variant (pT2N1aM0), in October 2005. Patient was in complete remission and was taking thyroid replacement therapy with 175 micrograms (*μ*g) of L-thyroxine. In February 2010, she presented with three-month history of headaches, vomiting, and gait ataxia. Pertinent neurological findings were dysmetria and dysdiadochokinesia. Stimulated serum thyroglobulin level was raised (51.7 *μ*g/L) with normal antithyroid antibodies. On I-131 whole body scintigraphy (WBS), no foci of abnormal tracer uptake were seen. Magnetic resonance imaging (MRI) of brain revealed a lobulated heterogeneous mass involving the medial left cerebellar hemisphere, measuring 4.0 × 3.5 × 2.5 cm. The mass was associated with surrounding edema, crossing the midline to the right cerebellar hemisphere and compressing the fourth ventricle (Figure 1). Differential diagnosis included a high grade glioma or metastasis. Subsequently, the patient underwent a left suboccipital craniectomy and complete resection of the cerebellar mass. Histopathology revealed papillary fronds with fibrovascular core lined by cuboidal tumor cells, with inclusion bodies (Figure 2). Immunohistochemistry was positive for cytokeratin (CK19), thyroid transcription factor (TTF-1), and thyroglobulin. These findings confirmed the diagnosis of metastatic PTC, follicular variant. The postoperative course was uneventful, and the patient was kept on follow-up, without any adjuvant treatment. In September 2012, she developed recurrence of cerebellar mass, along with new development of bilateral lungs and bone metastases, for which she was treated with palliative radiotherapy to posterior fossa and the right 7th rib followed by 150 mCi RAI ablation. Six months later, the patient died of progressive disease.

## 3. Case 2

**Figure 3:**
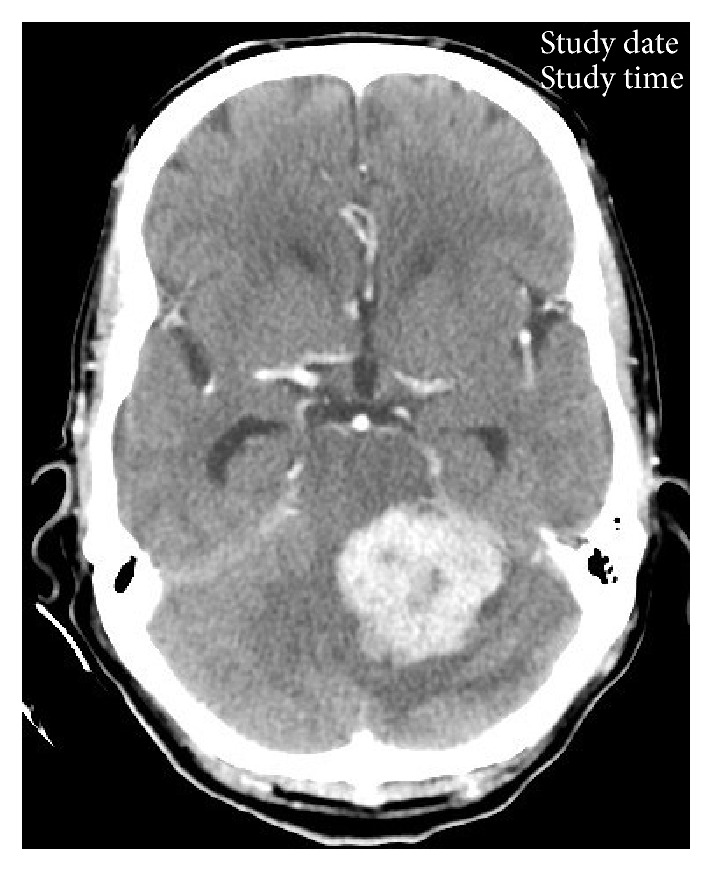
Computed tomography of brain (axial view) showing a 3.2 × 3.7 cm homogeneous contrast enhancing mass in the inferior vermis with extension into the left cerebellar hemisphere and brainstem causing hydrocephalus (the second case).

**Figure 4:**
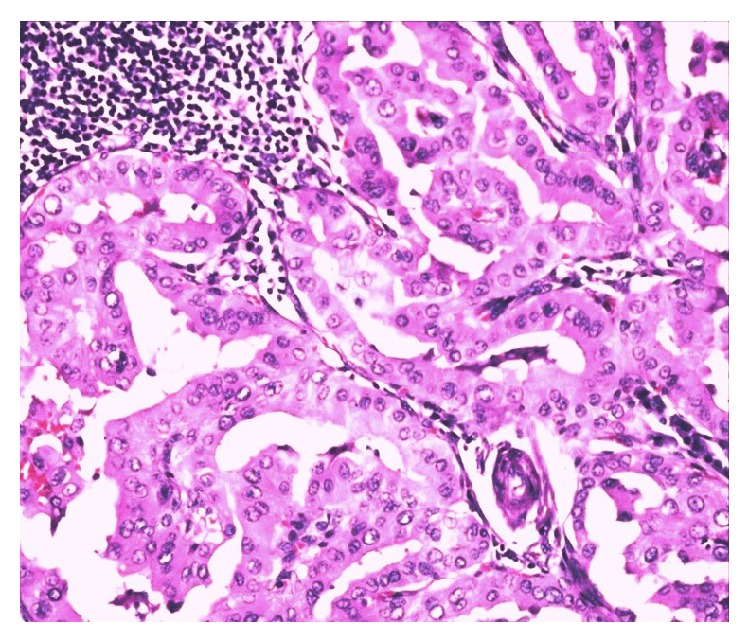
Hematoxylin and Eosin staining showing tall papillary cells (height at least twice or thrice their width), papillary thyroid carcinoma (the second case).

A 64-year-old Saudi male had been diagnosed with PTC in July 2009 and underwent total thyroidectomy. The tumor size was 2.5 × 3 cm and histopathological variant was tall cell PTC with extrathyroid extension; however no lymphovascular space invasion or lymph node metastases were noted. Final pathological TNM classification was T3N0 M0. After RAI ablation of 100 mCi, the patient was kept on follow-up. In August 2012, he started complaints of headaches and inability to walk. Serum TG level was 3290 *μ*g/L ↑. Neurological examination revealed bilateral papilledema and cerebellar signs on the left side. Computed tomography of brain showed a 3.2 × 3.7 cm homogeneous contrast enhancing mass in the inferior vermis with extension into the left cerebellar hemisphere and brainstem causing hydrocephalus (Figure 3). WBS showed intense uptake in both lungs. Patient underwent suboccipital craniectomy and subtotal resection of the mass. Histopathological examination of biopsied lesion revealed tall papillary cells, that is, height at least twice or thrice their width (Figure 4), and positive immunostaining for TTF-1 and CK19 confirmed the diagnosis of metastatic PTC, tall cell variant. Postoperatively, the lesion was treated with IMRT dose of 50 Gy in 25 fractions without any grade 3 toxicity, followed by RAI ablation dose of 150 mCi. At 30 months of follow-up, patient was found alive with stable lung metastasis, decreasing TG level (540 *μ*g/L) without any recurrence in the cerebellum.

## 4. Case 3

**Figure 5:**
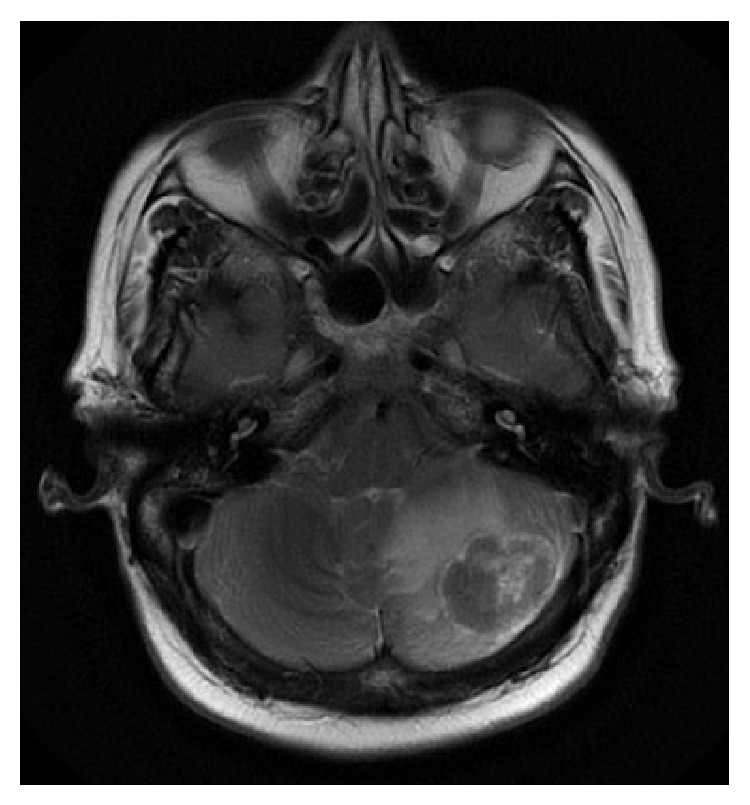
Magnetic resonance imaging (axial view) of brain showing a 4 × 4 cm heterogeneous mass in the left cerebellar hemisphere associated with edema (the third case).

**Figure 6:**
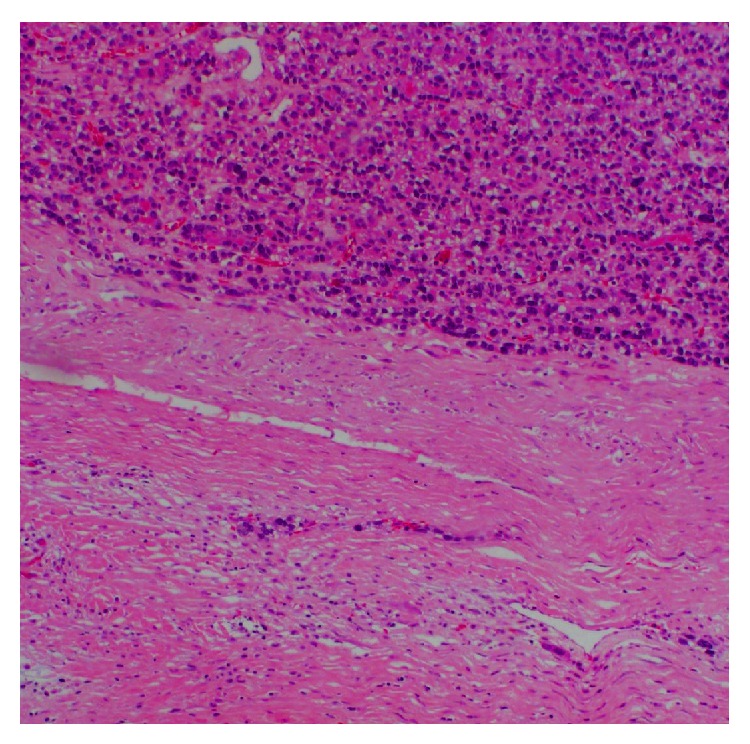
Hematoxylin and Eosin showing dark stained colloid (classic variant papillary thyroid cancer) (the third case).

A 65-year-old Saudi female underwent total thyroidectomy for multifocal PTC, classic variant (pT3N1bM0), followed by RAI ablation of 150 mCi in April 2008. Patient remained in complete remission and was on thyroid replacement therapy with 150 *μ*g L-thyroxine. In May 2013, she presented with 4-month history of vertigo, headaches, ataxic gait, and increased tiredness. Serum TG level was moderately elevated (77 *μ*g/L). MRI brain revealed a 4 × 4 cm heterogeneous mass in left cerebellar hemisphere associated with edema (Figure 5). Patient underwent suboccipital craniectomy and subtotal resection of the mass. Histopathological and immunostaining examination of biopsied lesion confirmed the diagnosis of metastatic PTC, classic variant (Figure 6). Postoperative IMRT of dose 50 Gy in 25 fractions was given to the tumor, without any grade 3 toxicity, followed by RAI ablation dose of 150 mCi. At 20 months of follow-up, patient was found alive without any evidence of recurrent or metastatic disease.

## 5. Discussion

CBM as initial manifestation of distant metastasis from PTC is rare. To date, only thirteen cases of CBM from PTC have been published (Table 1) [1–12]. In agreement with previously reported cases, CBM cases in our series were seen during the sixth and seventh decades of life with mean age of 67 years (range: 64–72), and mean duration between initial diagnosis and CBM was 49.6 months (range: 37–61) [1, 2, 4–6]. In our series, one patient with aggressive variant of PTC (tall cell) was identified. Tall cell variant PTC is known to have worse prognosis than classic PTC, and patients with these variants should be treated aggressively with thyroidectomy, neck dissection, and RAI, regardless of tumor size [13].

Diagnosis of CBM is challenging, as these lesions are often mistaken as chordoma, chondrosarcomas, meningioma, or schwannoma on CT and MRI imaging [4–7]. Histopathological diagnosis along with immunohistochemistry should be made in such cases for definitive diagnosis. All of our patients underwent suboccipital craniectomies, without any postoperative morbidity. However, complete surgical resection is often difficult in such cases because of presence of adjacent vital structures (brainstem, cochlea, and cranial nerves), risk of cerebrospinal fluid leak, and bleeding. In our series, only one patient had complete resection, followed by RAI ablation [6–12]. For such lesions, postoperative radiation therapy shall be offered, as one patient in our series developed cerebellar metastasis recurrence after surgery alone. With advent of novel techniques in radiation therapy (IMRT, protons, carbon ions, and stereotactic radiosurgery), it is now possible to give high dose to unresectable or residual tumor, to achieve better local control without any serious acute toxicities as seen in our patients [1–5].

Presence of CBM is generally considered as poor prognostic factor; however, in our patients, the median survival was 37 months (range: 21–60), which was found to be in agreement with literature [1–4, 6, 7].

In conclusion, cerebellar metastasis from PTC is a rare clinical entity and is often associated with hydrocephalus. Surgical resection followed by postoperative radiotherapy and RAI ablation is the treatment of choice. However, for such cases, multidisciplinary approach can prolong the disease-free and overall survival rates in PTC patients.

## Figures and Tables

**Table 1:** Reported cases of cerebellar metastasis from papillary thyroid carcinoma.

Reference	Age (years)/sex	Symptoms	Location	Treatment	Status	Follow-up
Tanaka et al. [1]	65/F	Lateral gazing nystagmus and slurred speech	Left cerebellar hemisphere with intratumoral hemorrhage	Subtotal resection Gamma knife surgery 15 Gy	Alive and disease-free	23 months

Cha et al. [2]	78/F	—	Left cerebellar hemisphere	Subtotal resection Gamma knife surgery 15 Gy	Alive and disease-free	36 months

Aguiar et al. [3]	33/F	Raised ICP	Right cerebellar hemisphere + cerebral lesions	Subtotal resection Radiation 40 Gy/20 fractions	At 12 months, developed frontal lobe metastasis Treated with surgery At 36 months, alive and disease-free	36 months

Lecumberri et al. [4]	65/F	—	—	Surgery and radiotherapy	At 48 months, developed cerebellar recurrence with intratumoral hemorrhage Treated with surgery	84 months

Pazaitou-Panayiotou et al. [5]	69/M	Dizziness, headaches, and ataxia	Right cerebellar hemisphere	Subtotal resection and RT 39 Gy/13 fractions	Dead with disease	4 months

Al-Dhahri et al. [6]	75/F	Dizziness, headache, and vomiting		Complete resection and RAI	—	—

Carcangiu et al. [7]	50/F	—	Right cerebellar hemisphere	Complete resection and RAI	Alive and disease-free	96 months

Honma et al. [8]	60/M	Incidental	Right cerebellar hemisphere	Surgery	Alive and disease-free	24 months

Xu et al. [9]	—	Incidental	Right cerebellar hemisphere	Surgery	—	—

Lin et al. [10]	Two cases	Headaches, ataxia, and vomiting	Case 1: bilateral cerebellar hemispheres with obstructive hydrocephalus Case 2: left cerebellar hemisphere	Complete resection	—	—

Jyothirmayi et al. [11]	—	Headache, ataxia	Left cerebellar hemisphere	Complete resection	—	—

Pacak et al. [12]	—	—	—	Complete resection	—	—
